# A novel insertion mutation in the cartilage-derived morphogenetic protein-1 (*CDMP1*) gene underlies Grebe-type chondrodysplasia in a consanguineous Pakistani family

**DOI:** 10.1186/1471-2350-9-102

**Published:** 2008-11-27

**Authors:** Sulman Basit, Syed Kamran-ul-Hassan Naqvi, Naveed Wasif, Ghazanfar Ali, Muhammad Ansar, Wasim Ahmad

**Affiliations:** 1 Department of Biochemistry, Faculty of Biological Sciences, Quaid-i-Azam University Islamabad, Pakistan

## Abstract

**Background:**

Grebe-type chondrodysplasia (GCD) is a rare autosomal recessive syndrome characterized by severe acromesomelic limb shortness with non-functional knob like fingers resembling toes. Mutations in the cartilage-derived morphogenetic protein 1 (*CDMP1*) gene cause Grebe-type chondrodysplasia.

**Methods:**

Genotyping of six members of a Pakistani family with Grebe-type chondrodysplasia, including two affected and four unaffected individuals, was carried out by using polymorphic microsatellite markers, which are closely linked to *CDMP1 *locus on chromosome 20q11.22. To screen for a mutation in *CDMP1 *gene, all of its coding exons and splice junction sites were PCR amplified from genomic DNA of affected and unaffected individuals of the family and sequenced directly in an ABI Prism 310 automated DNA sequencer.

**Results:**

Genotyping results showed linkage of the family to *CDMP1 *locus. Sequence analysis of the *CDMP1 *gene identified a novel four bases insertion mutation (1114insGAGT) in exon 2 of the gene causing frameshift and premature termination of the polypeptide.

**Conclusion:**

We describe a 4 bp novel insertion mutation in *CDMP1 *gene in a Pakistani family with Grebe-type chondrodysplasia. Our findings extend the body of evidence that supports the importance of *CDMP1 *in the development of limbs.

## Background

Grebe-type chondrodysplasia is a rare autosomal recessive syndrome characterized by severe acromesomelia, dwarfism, severe micromelia with increasing severity in a proximo-distal gradient and deformation of upper and lower limbs [1]. Radiologically, it is characterized by extremely short limbs, the legs being more severely affected than arms. The hands are extremely short with toe-like fingers, and the feet are in the valgus position [2]. Short and deformed middle long bones, fusion of carpal bones and several metacarpal and metatarsal, and absence of proximal and middle phalanges have been observed in the affected individuals. Other features include obesity and delayed mental development, but facial appearance and intelligence are normal with no vertebral abnormalities. Grebe-type chondrodysplasia is caused by mutations in the cartilage derived bone morphogenetic protein1 (*CDMP1*), located on chromosome 20q11.22. The *CDMP1 *gene is predominantly expressed in cartilaginous tissues of the developing long bones and the more distal elements of the appendicular skeleton that develop from the budding limb [3].

The *CDMP1 *has a murine homologue growth and differentiation factor-5 (*Gdf5*) and its absence leads to brachypodism in mice [4] and a number of skeletal malformation syndrome in humans including brachydactyly type A2 [5,6], brachydactyly type C (BDC) [7-10], fibular hypoplasia and complex brachydactyly (Du Pan syndrome) [11], Grebe-type chondrodysplasia [2,12-14], Hunter-Thompson type acromesomelic dysplasia [15], angel-shaped phalangeal dysplasia [16], multiple synostoses syndrome type 1 [17] and proximal symphalangism (SYM1) [5,10]. Recently, +104T/C (rs143383), a single nucleotide polymorphism in the untranslated region of the *CDMP1 *gene, was found to show significant association with osteoarthritis in Asian and European populations [18,19].

In the present study, we have ascertained a Pakistani family demonstrating autosomal recessive form of Grebe-type chondrodysplasia. DNA sequence analysis identified a novel four base insertion in *CDMP1 *gene.

## Methods

### Research subjects

**Figure 1:**
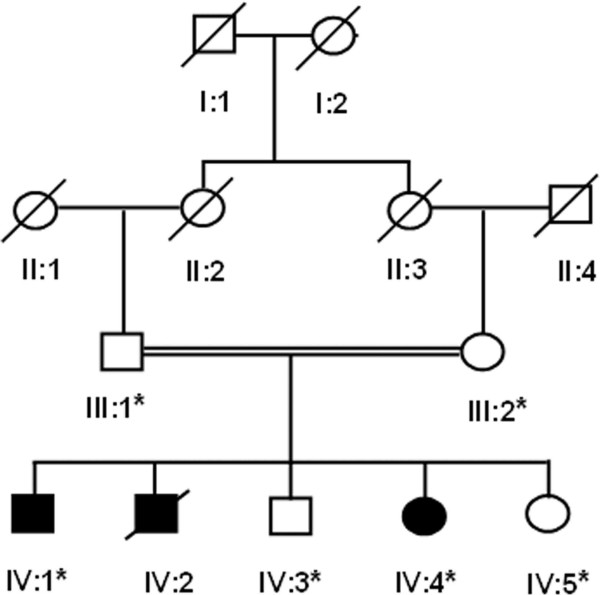
**Pedigree of a Pakistani family with Grebe type chondrodysplasia**. Open squares and circles represent unaffected males and females, respectively. Filled squares and circles represent affected individuals. Double lines between symbols are representative of consanguineous unions.

For the present study a Pakistani family with autosomal recessive Grebe-type chondrodysplasia (Fig. 1) was investigated. Prior to start of the study approval was obtained from the Quaid-i-Azam University Institutional Review Board. Informed consent was obtained from all subjects participating in the study. All affected and normal individuals underwent examination at Department of Diagnostic Imaging, Pakistan Institute of Medical Sciences, Islamabad. Blood samples were obtained from two affected (IV-1, IV-4) and four unaffected members (III-I, III-2, IV-3, IV-5) of the family.

### DNA extraction and genotyping

Genomic DNA was isolated from peripheral blood following a standard protocol and diluted to 40 ng/ul for amplification by polymerase chain reaction (PCR). The amplification of microsatellite markers was performed according to standard procedure in a total volume of 25 μl, containing 40 ng genomic DNA, 20 pmol of each primer, 200 μM of each deoxyribonucleoside triphosphates (dNTP), 1 unit of Taq DNA polymerase and 2.5 μl reaction buffer (MBI Fermentas, York, UK). The thermal cycling conditions used included 95°C for 1 min, followed by 35 cycles of 95°C for 1 min, 57°C for 1 min, 72°C for 1 min, and final extension at 72°C for 10 minutes. PCR products were resolved on 8% non-denaturing polyacrylamide gel, stained with ethidium bromide and genotypes were assigned by visual inspection.

Due to the autosomal recessive mode of inheritance and clinical features of the patients compatible with Grebe-type chondrodysplasia, the family was tested for linkage using microsatellite markers (D20S843, D20S195, D20S909, D20S865, D20S847, D20S834, D20S884) linked to *CDMP1 *gene on chromosome 20q11.22.

### Mutation detection

To search for an underlying mutation in *CDMP1 *gene, exons and splice junctions of the gene were amplified by PCR from genomic DNA using primers designed from intronic sequences of the gene. After PCR-amplified products were purified with commercially available kits (Marligen Biosciences, USA), sequencing was performed using DTCS-Quick start sequencing Kit (Beckman Coulter, USA) together with CQ8800 DNA sequencer (Beckman Coulter, USA). To amplify 440 bp PCR fragment encompassing part of exon 2 of the *CDMP1 *gene the following primers were used.

5'-GCTGGGAGGTGTTCGACATCT-3' (Exon 2, Sense)

5'-GCACTCCTGGAATCACAGAGG-3' (Exon 2, Antisense)

## Results

### Clinical findings

**Figure 2:**
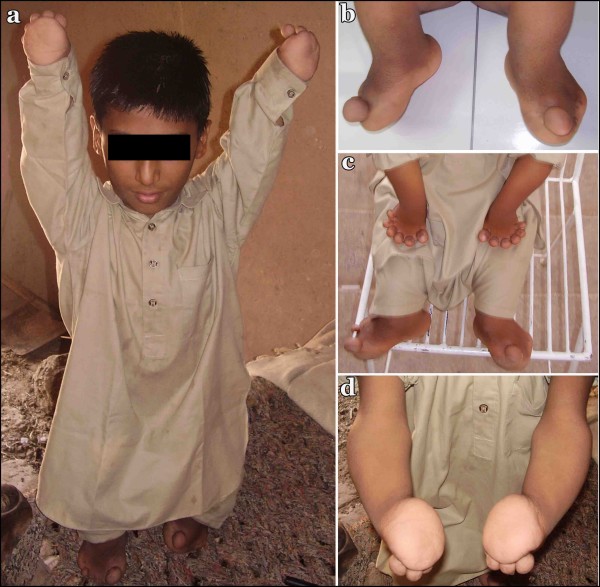
**Clinical features of the Grebe-type chondrodysplasia**. A 10 year's old affected member (IV-4) of the family (a), feet (b) and hands with hexadyctyly (c, d) of the same individual.

In the affected individuals of the family, presented here, abnormal findings were limited to the limbs (Fig 2). In the upper limbs, both arms were short, with forearms relatively shorter and bowed. The hands were very short and the fingers were replaced by globular appendages. In the lower limbs, the proximal segments were short and the middle segments were relatively shorter. In the feet the toes were small and overriding with limited movement. Polydactyly was observed in both hands.

**Figure 3:**
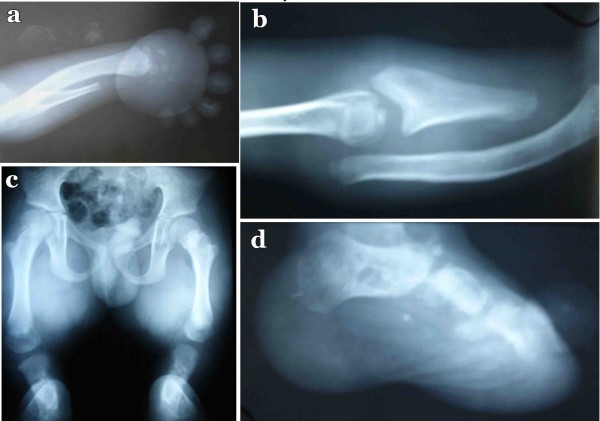
**Radiographs of the 10 year's old affected boy (IV-4) with Grebe type chondrodysplasia**. Radiographs of hand (a), arm (b), pelvis (c) and foot (d).

Radiographic examination of the upper limbs revealed that radius was malformed and the radial head was displaced upward out of the elbow joint. Pruning of the ulna was noted with sharp distal edges (Fig 3). Carpal bones were distorted and hypoplastic, and metacarpals were completely missing. Distal phalanges were present however proximal and middle phalanges were absent. In the lower limb femoral and tibial bones were short with the absence of tibial plateau. Fibulas were completely absent. Thoracic spines, bones and skull appeared normal.

### Genotyping

To identify the underlying autosomal recessive Grebe-type chondrodysplasia in the family, genotyping was performed with microsatellite markers closely linked to *CDMP1 *gene on chromosome 20q11.22. The affected members of the family were homozygous for four markers (D20S843, D20S195, D20S865, D20S847), thus suggesting linkage of the family to *CDMP1 *gene.

### Mutation analysis

**Figure 4:**
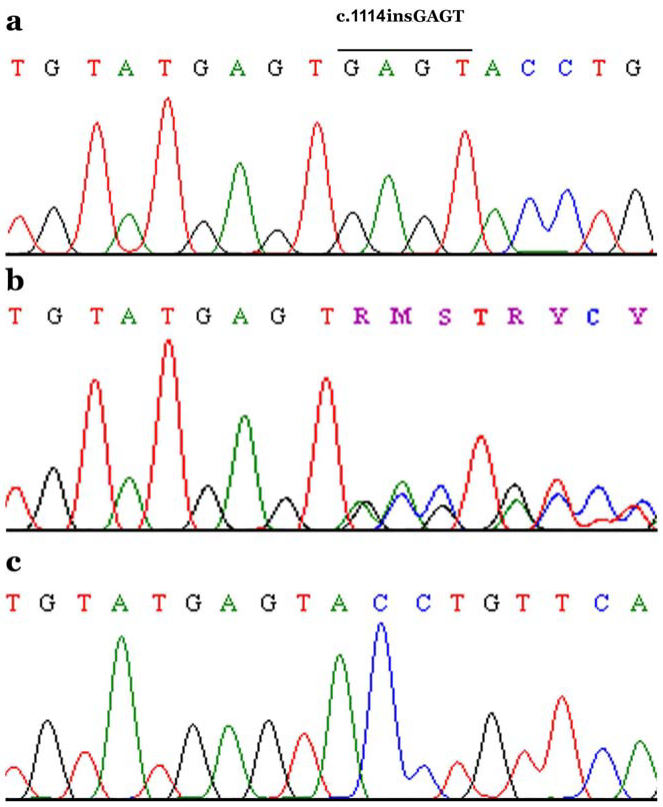
**Sequence analysis of the *CDMP1 *gene mutation**. DNA sequence of exon 2 of *CDMP1 *gene from (a) an affected individual (b) a heterozygous carrier and (c) a control individual. The bar in panel (a) represents the sequence that was inserted in the homozygous state in the affected individuals.

The entire coding portion and intron-exon boundaries of *CDMP1 *gene was sequenced in 6 individuals including two affected (IV-1 and IV-4) of the family. Sequence analysis of exon 2 of the *CDMP1 *gene from affected individual revealed a 4-bp insertion starting at nucleotide position 1114 (1114insGAGT) (Fig. 4a) resulting in immediate stop codon. This insertion was present in the heterozygous state in obligate carriers III-1 and III-2 (Fig. 4b). The mutation was not identified in normal individuals of the family (Fig. 4c). To ensure that the mutation does not represent a neutral polymorphism in this population, a panel of 40 unrelated unaffected ethnically matched control individuals was screened for the mutation and it was not identified outside the family.

## Discussion

In the present investigation we have described a second Pakistani family with Grebe-type chondrodysplasia. The phenotype of the present family shares clinical and radiographic features with three families with Grebe-type chondrodysplasia reported earlier [2,12,13]. The Pakistani family, presented here, displayed severe shortening of both upper and lower limbs. Hexadactyly was observed in both hands of the affected individuals. Radiographic examination of the affected individuals showed severely deformed tibiae, ulnae and radii. Fibulae were completely absent in the affected individuals. Physically apparent clinical defects were not seen in any of the obligate carriers of the family. Lack of availability of skeletal radiographs of the obligate carriers makes it difficult to exclude subtle skeletal deformities which were not detected by physical examination.

To date, eighteen pathogenic mutations in the *CDMP1 *gene have been identified including three in patients affected with Grebe type Chondrodysplasia [2,12,13]. Cartilage-derived morphogenetic protein-1 (CDMP1), a secreted protein, is a member of the bone morphogenetic protein (BMP) family and the transforming growth factor-beta (TGF-β) superfamily [3,20,21]. The CDMP1 precursor polypeptide contains 501 amino acids with a RRKRR polybasic proteolytic processing site at amino acids 377–381 and seven highly conserved cystiene at its C-terminus. It forms homodimers or heterodimers with other BMP partners linked by a single inter-chain disulfide bond. These dimers undergo proteolysis, producing an active mature CDMP1 dimer that is secreted from the cell [20]. The CDMP1 is a ligand of BMP receptors (Types 1 and 2) resulting in an activation of BMPR1B by transphosphorylation through BMPR2. The transmembrane serine/threonine kinase receptor BMPR1b subsequently activates the SMAD-dependent pathway and/or the p38 MAP kinase pathway. Both signal cascades regulate the transcription of specific target genes that are involved in bone formation [22-24].

In the family with Grebe type Chondrodysplasia, presented here, sequence analysis of the *CDMP1 *gene detected a homozygous 4-bp insertion mutation (1114insGAGT) in both the affected individuals. This mutation is predicted to produce a truncated protein consisting of 371 amino acids and lacking an active domain, which is expected to abolish the subsequent signaling pathways in CDMP1 target cells.

The CDMP1/GDF5 is expressed predominantly in developing limbs at joint interzones, and is known to play an important role in joint formation [2,3]. The expression pattern of GDF5 at joint interzones suggested a possible role for CDMP1/GDF5 in establishing boundaries between skeletal elements [25]. As suggested by Francis et al. [26] that the interzones can be regarded as a signaling center, regulating chondrocytes proliferation and differentiation and orchestrating joint formation. Absence of CDMP1/GDF5 results in abnormal joint formation and ligament defects.

## Conclusion

We have reported the fourth mutation in *CDMP1 *gene, which results in autosomal recessive Grebe-type chondrodysplasia. The study presented here confirms that CDMP1 plays an important role in the regulation of the development skeleton, and its absence does not impair other development processes.

## Abbreviations

*CDMP1*: cartilage-derived morphogenetic protein 1; *GDF5*: growth and differentiation factor 5; BMP: bone morphogenetic protein; BMPR: bone morphogenetic protein receptor; TGF: transforming growth factor; DTCS: dye terminator cycle sequencing.

## Consent

Written consent was provided by father of the patients for publishing photographs and other material.

## Competing interests

The authors declare that they have no competing interests.

## Authors' contributions

SB participated in the design of the study, performed PCR, gene sequencing and manuscript writing. SKN, NW, GA, MA studied family, collected blood samples and extracted DNA. WA analyzed the data, participated in manuscript preparation and collected funds for the study. All authors read and approved the final manuscript.

## Pre-publication history

The pre-publication history for this paper can be accessed here:


